# Supplementary material for: Pacemaking function of two simplified cell models
Source: PLoS One. 2022 Apr 11;17(4):e0257935. doi: 10.1371/journal.pone.0257935 (PMC9000119; doi:10.1371/journal.pone.0257935)
Supplement: S1 Fig — Comparison of the accuracy of Forward Euler and Backward Euler methods. The MATLAB and CellML codes are available at https://github.com/mryzhii/Simplified-pacemaker-cell-models. (PDF) [file pone.0257935.s001.pdf]

S1 Fig. Action potentials and phase portraits of the pacemaking variants of Aliev-Panfilov (pAP) and Corrado (pCN) models.

Comparison of the accuracy of Forward Euler (FE) and Backward Euler (BE) methods with different time steps dt. The MATLAB and CellML codes are available at GitHub public repository: https://github.com/mryzhii/Simplified-pacemaker-cell-models.

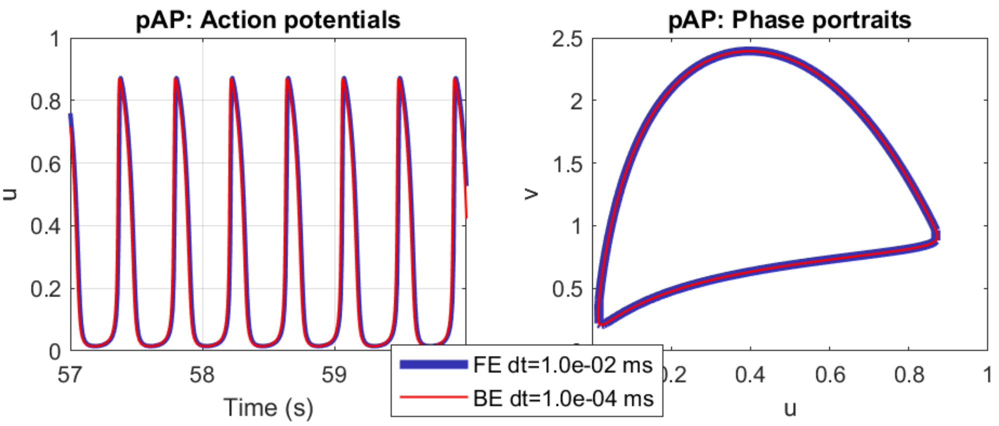

Pacemaking Aliev-Panfilov model: Backward Euler / Forward Euler:
BE: dt_backward = 0.00010, Period_BE = 0.42523, Freq_BE = 2.3516, Ampl_BE = 0.8566
FE: dt_forward = 0.01000, Period_FE = 0.42531, Freq_FE = 2.3512, Ampl_FE = 0.8566
L_2 relative norm = 0.00048090 / 0.0481%
L_inf relative norm = 0.00290912 / 0.2909%
Frequency_BE-Frequency_FE = 3.94950e-04, d_Frequency_rel = 1.67946e-04 / 0.017%
sim_time_BE/sim_time_FE = 263.79

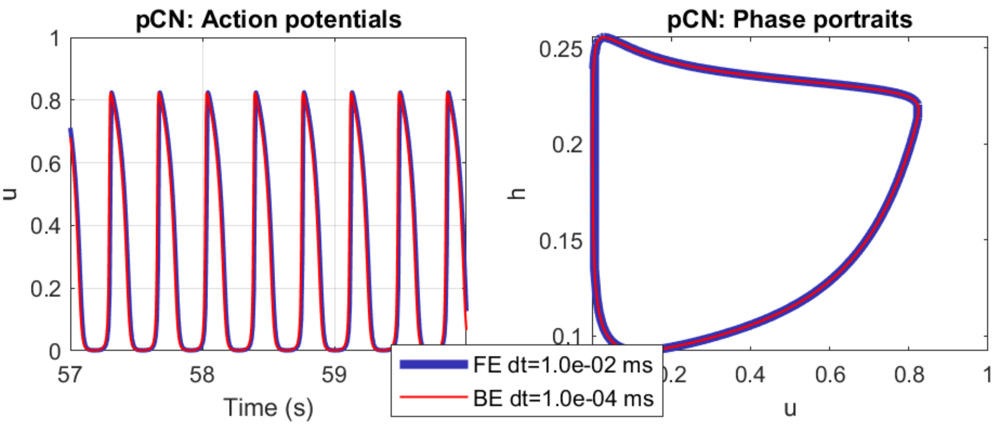

Pacemaking Corrado-Niederer model: Backward Euler / Forward Euler:
BE: dt_backward = 0.00010, Period_BE = 0.3655, Freq_BE = 2.7356, Ampl_BE = 0.8229
FE: dt_forward = 0.01000, Period_FE = 0.3656, Freq_FE = 2.7352, Ampl_FE = 0.8230
L_2 relative norm = 0.00068111 / 0.0681%
L_inf relative norm = 0.00619355 / 0.6194%
Frequency_BE-Frequency_FE = 4.28881e-04, d_Frequency_rel = 1.56775e-04 / 0.016%
sim_time_BE/sim_time_FE = 132.01
