# Supplementary material for: Pacemaking function of two simplified cell models
Source: PLoS One. 2022 Apr 11;17(4):e0257935. doi: 10.1371/journal.pone.0257935 (PMC9000119; doi:10.1371/journal.pone.0257935)

S2 Fig. Shifting of the POP and MDP levels in the pAP and pCN models.

1) Modified pAP/AP model. The parameter u_M changes the MDP (rest) value, and u_P changes the amplitude of the transmembrane potential (POP value).

$$\frac{\partial u}{\partial t} = c_t[k(u - u_M)(u + b_{AP})(u_P - u) - (u - u_M)v]$$

$$\frac{\partial v}{\partial t} = c_t \varepsilon[-v - k(u - u_M)(u - a - u_P)]$$

$$\varepsilon = \varepsilon_0 + v\mu_1/(u - u_M + \mu_2)$$

	k	a	μ_1	μ_2	b_{AP}	u_M	u_P
Original pAP model	8	0.13	0.2	0.3	0.02	0	1.0
Modified pAP model					0.22	-0.2	0.8

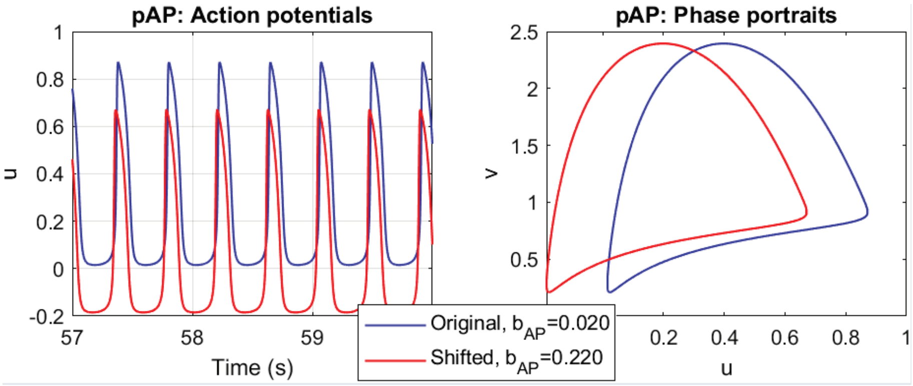

2) Modified pCN/CN model. The parameter u_M changes the MDP (rest) value, and u_P changes the amplitude of the transmembrane potential (POP value).

$$\frac{\partial u}{\partial t} = h(u - u_M)(u + b_{CN})(u_P - u)/\tau_{in} - (1 - h)(u - u_M)/\tau_{out}$$

$$h_\infty = 0.5\left[1 - \tanh\left((u - u_M - \mu_{gate})/u_s\right)\right]$$

	τ_{in}	τ_{out}	τ_{open}	τ_{close}	u_s	u_{gate}	b_{CN}	u_M	u_P
Original pCN model	0.3	6.0	120	150	0.15	0.01	0.30	0	1.0
Modified pCN model							0.50	-0.2	0.8

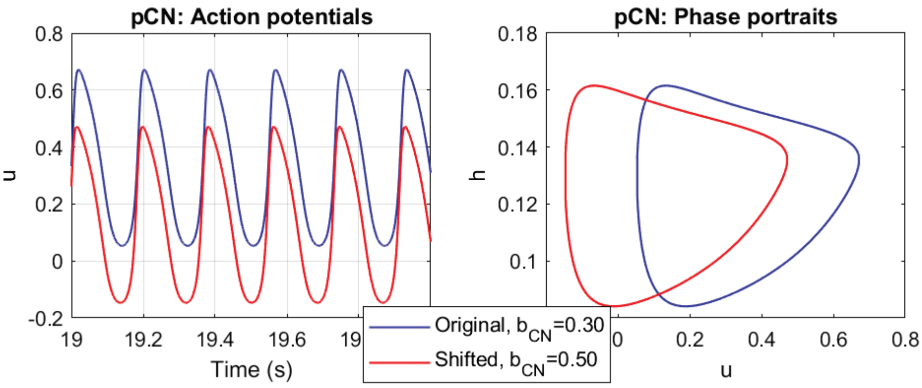

Supplement: S2 Fig — (PDF) [file pone.0257935.s002.pdf]
