# Supplementary material for: Pacemaking function of two simplified cell models
Source: PLoS One. 2022 Apr 11;17(4):e0257935. doi: 10.1371/journal.pone.0257935 (PMC9000119; doi:10.1371/journal.pone.0257935)
Supplement: Attachment — Submitted filename: Rebuttal_letterR2.pdf [file pone.0257935.s012.pdf]

Summary of modifications and responses to reviewers' comments (R2)

Dear Dr. Guerrero-Hernandez,

We are very grateful to you and the reviewers for their consideration and for pointing out ways to improve the technicality and readability aspects of our manuscript. We have prepared a revised version of the paper with changes and additions suggested by the reviewers.

Regarding the reviewers' valuable comments, the following changes have been made in the paper:

- We have renamed the Section "General remarks" to "Limitations and general remarks" and added the text (lines 444-474).
- We have added Supplementary S2 Fig describing methods of the resting (MDP) and amplitude/peak (POP) levels shifting in the pAP and pCN models, which can be useful for simulations with resting and/or peak level differences between cell types.

In the specific responses that follow, reviewers' comments appear in italic.

Response to Reviewer 3:

We thank Reviewer 3 for the comments, which helped us improve the manuscript. These comments are implemented in the revised manuscript. The following provides our response to the comments.

In this version of the paper, the authors have tried to make the examples more realistic and have, for the most part succeeded. However, their type 2 SAN simulations look odd. That put a passive barrier around their SAN instead of an insulating one. As such, electrotonic interactions occur across the barrier which affects propagation in the vicinity. This should not occur.

The authors should list the limitations of their model. While such a model can be useful at times, it is also important to say when it cannot. For example, how limited is the morphology? Can it support a bursting mode on top of the plateau? Since voltage is normalized, it appears that resting level differences cannot be incorporated.

We have renamed the Chapter "General remarks" to "Limitations and general remarks", added the text below (lines 444-474), and added Supplement S2 Fig describing methods for shifting of the resting (MDP) and amplitude/peak (POP) levels.

"…The results in Figs 8 and 9 are presented for illustration purposes and not aimed at a detailed study of these organs. In particular, in the SAN type 2 model (Fig 3B), the tissue of the borders surrounding the pacemaking area is of passive type, blocking the spread of excitation [28]. Such tissue, being not exactly of an electrically insulating type [59, 60], causes electrotonic interactions with the surrounding SAN and atrial tissues. Better selection of the boundary tissue properties can be beneficial for more realistic simulations of the SAN and its exit pathway functions. Moreover, additional tuning of the parameters to clinical data might be necessary for accurate simulations, in particular, to construct patient-specific models. This can be realized by applying, for example, a robust and clinically tractable protocol and fitting algorithm [19] for characterizing cardiac electrophysiology properties by simplest two-variable cell models, such as the pAP-AP and pCN-CN coupled systems.

For the simulations where the differences of resting and/or peak levels are necessary, the MDP and POP values of the considered models can be modified. The addition of a constant to the term u and the replacement of unity in the term $(1 - u)$ allow shifting of the MDP and POP levels, respectively, though the exact resulting values of the latters cannot be determined directly (see Supplement S2 Fig for the details). Also, such modification may require an adjustment of the intrinsic frequencies (for example, with the parameters b_{AP} and b_{CN}). The above modifications of the MDP and POP together with modulation of the intrinsic frequency with the parameters b may allow simulation of a kind of tonic bursting [29] - a firing behavior in which a neuron cell fires a certain

number of spikes on the top of the plateau and is silent for a certain amount of time. Though, compared to the variety of specific neuronal cell models [29], the pAP and pCN models may not be the best choice for modeling of neuronal systems.

Another well-known disadvantage of the phenomenological models like pAP-AP and pCN-CN is their limited ability to represent action potential morphology under varying physiological conditions, e.g., the effect of variation of concentration of particular ions, which is necessary for simulations of complex cardiac diseases such as ion channelopathies. However, …"

Response to Reviewer 4:

We thank Reviewer 4 for useful and insightful comments, which helped us improve the manuscript.

Sincerely yours,

Maxim Ryzhii
Complex Systems Modeling Laboratory, University of Aizu, Japan

Elena Ryzhii
Department of Anatomy and Histology, Fukushima Medical University, Japan
